# Role of 18F-FDG PET/CT in patients affected by pulmonary primary lymphoma

**DOI:** 10.3389/fonc.2022.973109

**Published:** 2022-09-14

**Authors:** Ying Peng, Wanling Qi, Zhehuang Luo, Qingyun Zeng, Yujuan Huang, Yulu Wang, Amit Sharma, Ingo G. H. Schmidt-Wolf, Fengxiang Liao

**Affiliations:** ^1^ Department of Cardiology, Jiangxi Provincial People’s Hospital, The First Affiliated Hospital of Nanchang Medical College, Nanchang, China; ^2^ Department of Nuclear Medicine, Jiangxi Provincial People’s Hospital, The First Affiliated Hospital of Nanchang Medical College, Nanchang, China; ^3^ Clinical Laboratory, The First Affiliated Hospital of Nanchang University, Nanchang, China; ^4^ Department of Integrated Oncology, Center for Integrated Oncology (CIO), University Hospital Bonn, Bonn, Germany; ^5^ Department of Neurosurgery, University Hospital Bonn, Bonn, Germany

**Keywords:** FDG (18F-fluorodeoxyglucose)-PET/CT, primary pulmonary lymphoma (PPL), metabolic characteristic, Chinese patients, prospective

## Abstract

**Background:**

Primary pulmonary lymphoma (PPL) is defined as clonal abnormal hyperplasia of lung parenchyma or bronchial lymphoid tissue originating from bronchial mucosal tissue. However, PPL is rare, which accounts for approximately 3-4% of extraneurotic lymphomas and 0.5-1% of all primary tumors in the lung. Owing to the lack of any typical clinical symptoms and radiological features, it is challenging to accurately diagnose PPL, which affects its clinical management and prognosis. Considering this, herein, we aim to raise awareness of this disease and help physicians understand the role of 18F-FDG PET/CT in the diagnosis of PPL.

**Method:**

A retrospective analysis was performed on the clinical and 18F-FDG PET/CT imaging data of 19 patients diagnosed with PPL by biopsy pathology at our hospital from April 2014 to December 2021.

**Results:**

Of the 19 PPL patients, 15 patients showed clinical symptoms with the most common being fever and cough. In addition, there were 4 cases that had no clinical symptoms, and all of them were MALT lymphoma. In fact, 16 patients were misdiagnosed as lobar pneumonia, lung cancer, tuberculosis, and diffuse interstitial inflammation, representing a misdiagnosis rate of 84.2%. Also, 73.7% were MALT lymphomas, representing the most common pathological pattern, along with 3 DLBCL and 2 T-cell lymphomas. With reguard to CT signs, the air-bronchial sign was found to be the most common, followed by the halo sign and the collapsed leaf sign. On the basis of the predominant radiologic features, lesions were categorized as pneumonic consolidation, nodular/mass type, diffuse interstitial type, and mixed type. The average SUVmax of lesions was 7.23 ± 4.75, the ratio of SUVmax (lesion/liver) was 3.46 ± 2.25, and the ratio of SUVmax (lesion/mediastinal blood pool) was found to be 5.25 ± 3.27. Of interest, the different pathological types of PPL showed different values of 18F-FDG uptake. The 18F-FDG uptake of DLCBL was the most prominent with a SUVmax of 15.33 ± 6.30 and was higher than that of MALT lymphoma with a SUVmax of 5.74 ± 2.65. There appeared similarity in 18F-FDG uptake between MALT lymphoma and T-cell lymphoma. For the SUVmax of lesion, we found statistical significance between MALT lymphoma and DLCBL (P value<0.001). In addition, we also found statistical significance (P value < 0.05) in SUVmax of lesions between pneumonic consolidation type and nodal/mass type, I stage, and other stages.

**Conclusions:**

On 18F-FDG PET/CT images, certain features of PPL morphology and metabolism can be identified that may contribute to a better understanding of this disease. In addition, 18F-FDG PET/CT whole-body imaging has the potential to refine the staging of PPL. Most importantly, functional 18F-FDG PET/CT imaging can readily reflect tumor cell activity, thus allowing for the selection of an optimal biopsy site.

## 1 Introduction

Pulmonary lymphoma (PL) accounts for about 25-40% of all lymphomas, and most of them are secondary pulmonary lymphoma (SPL), i.e., extrapulmonary lymphoma that are transmitted into the blood or mediastinal lymph nodes and directly infiltrated into the lungs (1). Compared to other extranodal lymphomas, Primary pulmonary lymphoma (PPL) is rare, accounting for about 3-4% of extraneurotic lymphoma and 0.5-1% of all primary tumors in the lung, and the incidences of PPL in non-Hodgkin’s lymphoma(NHL) are reported to be less than 1% (1, 2). PPL is primarily defined as clonal abnormal hyperplasia of pulmonary parenchyma or bronchial lymphoid tissue with or without enlargement of hilar lymph nodes, and without evidence of extrapulmonary lesions at the onset or within three months of diagnosis (3).

PPL originates from tissues related to the bronchial mucosa, invades mainly the interstitium of the lung and bronchial submucosa tissues, and grows along the bronchial submucosa, usually not causing bronchial obstruction (4). In the early stages, there are usually no obvious clinical symptoms, and it is also not specific when clinical symptoms may appear (5). However, the foremost symptoms include fever, cough, weight loss, fatigue, and dyspnea. Some patients even have no clinical symptoms, especially in the lung mucosa-related lymphoid tissue lymphoma. Since the clinical symptoms and radiological features are atypical, the disease is difficult to diagnose and is often misdiagnosed as pneumonia, lung cancer, tuberculosis, etc., which affects the clinical treatment and prognosis (6, 7). Currently, there are only a few studies on PPL in the literature, mainly focusing on clinical and CT morphological reports, with small sample sizes and a lack of systematic investigations. Moreover, there are fairly rare studies on the metabolic characteristics of PPL in 18F-FDG PET/CT, with occasional case reports (8).

Considering this, herein, we performed a retrospective analysis on the clinical and 18F-FDG PET/CT imaging data of 19 patients diagnosed with PPL by biopsy pathology. We primarily summarize certain features of PPL morphology and metabolism identified using 18F-FDG PET/CT images that may contribute to a better understanding of this disease.

## 2 Data and methods

### 2.1 Clinical data

The clinical and 18F-FDG PET/CT imaging data from 19 patients diagnosed with PPL by biopsy pathology at our hospital between April 2014 and December 2021 were retrospectively analyzed. The diagnostic criteria used in screening of PPL: (a) diagnosis of lymphoma by lung biopsy or surgical pathology of lung tissue; (b)no previous history of extrapulmonary lymphoma; (c) no lymphoma was found outside the lung within 3 months after diagnosis (2, 3).

Among the 19 patients with PPL, there were 11 males and 8 females aged 25-80 years old, with a median age of 56 years old and an average age of 59.1 ± 15.7 years old. The clinical data of the study included: (a) Clinical symptoms: fever, respiratory symptoms (cough, chest pain, dyspnea), fatigue, no symptoms, etc.(b)Clinical staging [according to Ferraro et al. revised Ann Arbor staging criteria (9)]: Stage IE, unilateral or bilateral lung lesions; Stage IIE, lung lesions and involvement of the hilar and/or mediastinal lymph nodes; Stage IIEW, lung lesions and involvement of the adjacent chest wall or diaphragm; StageIII, pulmonary lesions and involvement of the subphrenic lymph nodes; Stage IV, diffuse lesions in both lungs.

### 2.2 Imaging method

The fasting blood glucose (< 11.1 mmol/L) was measured in all patients after more than 6 hours of fasting before imaging. Next, 18F-FDG was intravenously injected at a dose of 0.10-0.14 mCi/kg. Patients were allowed to lie down and rest for about approximately 40-60 minutes prior to the CT scan. The scan area extended from the top of the skull to the mid-thigh, with a slice spacing and thickness of 3.75 mm. Finally, 18F-FDG PET images were acquired in the same range in 3D mode. The acquisition time was 3 min/bed, and a total of 6-8 beds were acquired. Following the iterative reconstruction of the images whole-body 3D MIP maps and transverse, coronal, and sagittal images were obtained. After 18F-FDG PET/CT acquisition, a diagnostic thin-slice CT scan of the lung (120 V, 250 mA) with a thickness of 1.25 mm from the thoracic inlet to the base of the lung was performed separately. The 18F-FDG was prepared, synthesized, and tested using a GE MINIstrance cyclotron, tracerlab FX chemistry auto synthesizer, and discovery STE, respectively. The radiological purity of the radiopharmaceuticals was greater than 95%.

### 2.3 Imaging analysis

The 18F-FDG PET/CT images were evaluated separately by two senior physicians or associate chief physicians of the nuclear medicine department. In case of disagreement, consensus was reached by consultation, or the final result was assessed by the higher-level physician.

#### 2.3.1 Qualitative analysis

The location, distribution, density and margins of the lesions were observed, and the CT signs of the lesions, such as air bronchial signs, halo signs, transfoliar signs, cystic lesions, bronchiectasis, and pleural involvement were analyzed. The extent and distribution of FDG uptake and extrapulmonary involvement were also studied Higher uptake in the lesion compared to liver was considered abnormal.

Definition of CT signs: (a) Air bronchial sign-refers to normal aerated bronchial passage within the lesion; (b) Halo sign- refers to the density shadow of ground glass around the lesion; (c) Translobular sign- refers to the distribution of lesions invading pulmonary fissure and translobar; (4) Pleural involvement includes localized or diffuse pleural thickening, with or without pleural effusion.

#### 2.3.2 Quantitative analysis

The number of lesions was recorded on CT, and the size of lesions and lymph nodes were measured, including maximum diameter and minimum diameter. The maximum standard uptake value (SUV = local tissue radioactivity concentration (kBq/kg)/(injected dose (kBq)/body weight (kg)) of lesions, liver and mediastinal blood pool was measured by PET, and the proportion was calculated. A SUVmax of the lesion greater than that of liver was considered abnormal, and hilar/mediastinal lymph node exceeding 10-mm was considered as lymph node enlargement. The portion of the hypermetabolic lesion on PET was outlined with a 10-mm circle to delineate the Region of Interest(ROI) (10). And the SUVmax of the lesion for each pathological uptake and the SUVmax of liver and blood pool were measured. Subsequently, we calculated the the SUVmax ratio of lesion to liver and lesion to blood pool.

### 2.4 Statistical analysis

Demographic and clinical data were presented as means, medians, minimum or maximum, frequencies and percentage. Continuous data between two different groups were compared using Student’s t-test, whereas, categorical data were compared using χ2 test. The statistical analysis was carried out with SPSS 23.0 version. P values less than 0.05 were considered statistically significant. The relationship between semi-quantitatively features (SUVmax, lesion-to-liver SUVmax ratio and lesion-to-blood pool SUVmax ratio) and the morphological parameters were calculated using Anova test and independent t-test.

## 3 Result

### 3.1 Clinical manifestation

The clinical manifestation of the 19 PPL patients is shown in **Table 1**. Among the 19 PPL patients, there were 11 males and 9 females, aged from 25 to 81 years old with average age of 59.1 ± 15.7 years old. Fifteen patients had clinical symptoms, with fever and cough being the most common in 8 cases each. Other clinical symptoms such as dyspnea (6 cases), chest pain (4 cases) and fatigue (4 cases) were also included. In addition, there were 4 cases that had no clinical symptoms, and all of them were MALT lymphoma. In the study, 16 patients were misdiagnosed as lobar pneumonia (6 cases), lung cancer (4 cases), tuberculosis and diffuse interstitial inflammation (3 cases), representing a misdiagnosis rate of 84.2%. All the 19 patients were pathologically confirmed by biopsy, including 10 cases with percutaneous lung puncture biopsy, 4 cases with fibrobronchoscopy biopsy, and 5 cases with surgical resection biopsy.

**Table 1:** Clinical findings in 19 patients with PPL.

	No.(%)
Gender	
Male	11(57.9%)
Female	8 (42.1%)
Age (years)	
Average	59.1 ± 15.7
Range	25˜81
Common symptoms	
Fever	8
Cough	8
Dyspnea	6
Chest pain	4
Fatigue	3
No comfortable	4
Misdiagnosis	
Lobar pneumonia	6 (31.6%)
Lung cancer	4 (21.1%)
Tuberculosis	3 (15.8%)
Diffuse interstitial pneumonia	3 (15.8%)
Confirmed by	
Percutaneous lung biopsy	10 (52.6%)
Bronchoscopic biopsy	4 (21.1%)
Surgical excision biopsy	5 (26.3%)

### 3.2 Imaging findings

**Figure 1:**
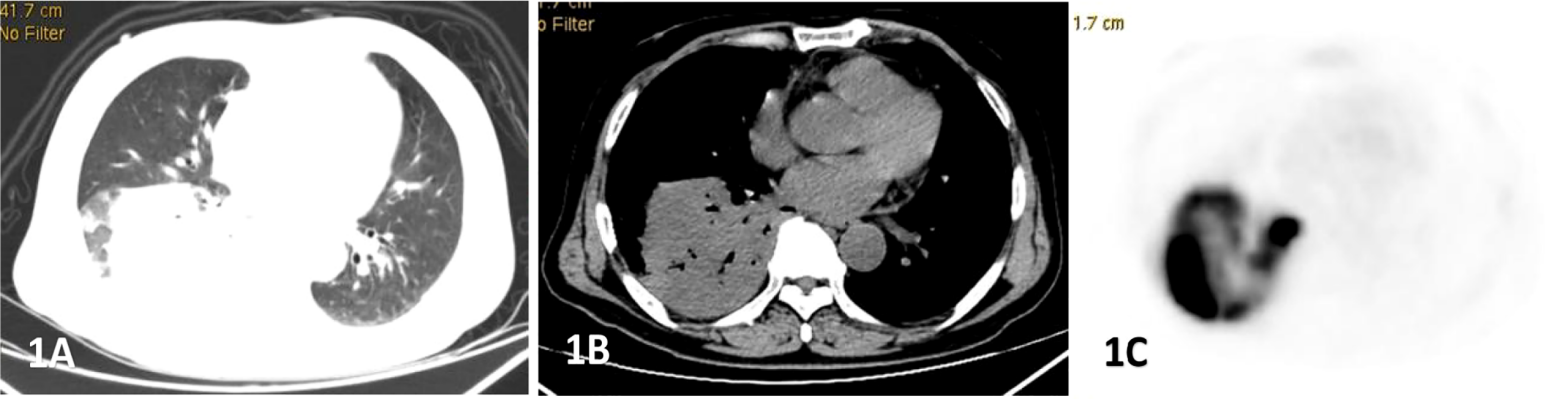
Male, 69 years old, fever, cough, dyspnea for 3 months. **(A–C)** showed lung, mediastinal CT scan and PET transection images respectively. Soft tissue mass shadow was observed in the lower lobe of the right lung, and aerated bronchial shadow was observed in the lesion, showing cross-lobe growth with a size of about 81×77mm.The FDG uptake of the lesion was significantly increased, with an SUVmax of 22.6. Percutaneous lung biopsy was conformed diffuse large b-cell lymphoma(DLBCL).

**Figure 2:**
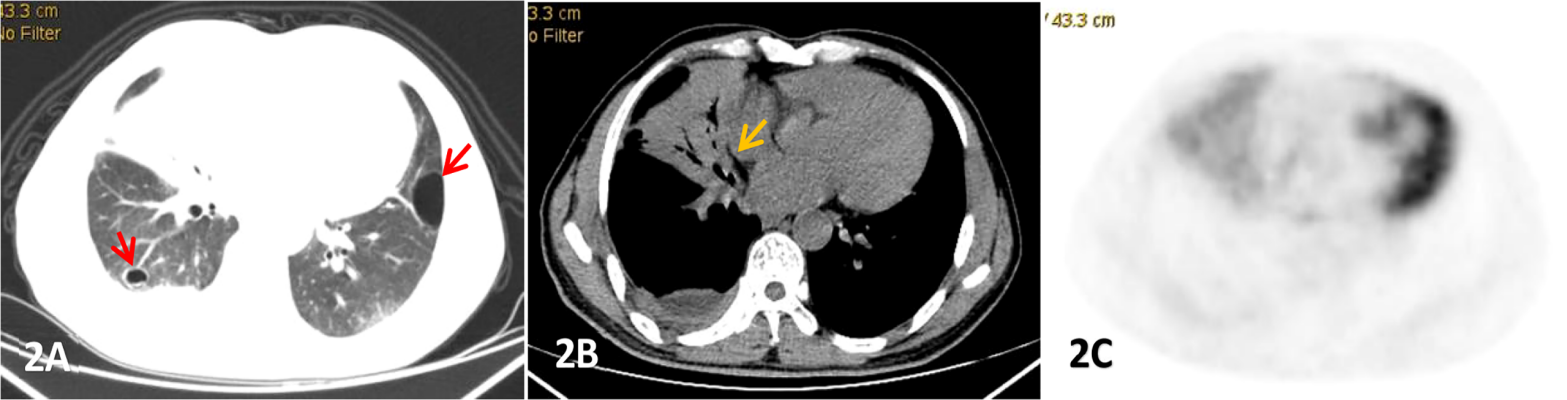
Male, 53 years old, cough and expectoration accompanied by right lower chest pain for more than 2 months, **(A-C)** were lung, mediastinum CT transection scan and PET transection image respectively,. Large consolidation shadow was showed in the middle lobe of the right lung with air bronchial signs(yellow arrow),which FDG uptake increased with an SUVmax of 3.83, right pleural thickening and pleural effusion, and cystic lesions(red arrow) were also observed in both.

**Figure 3:**
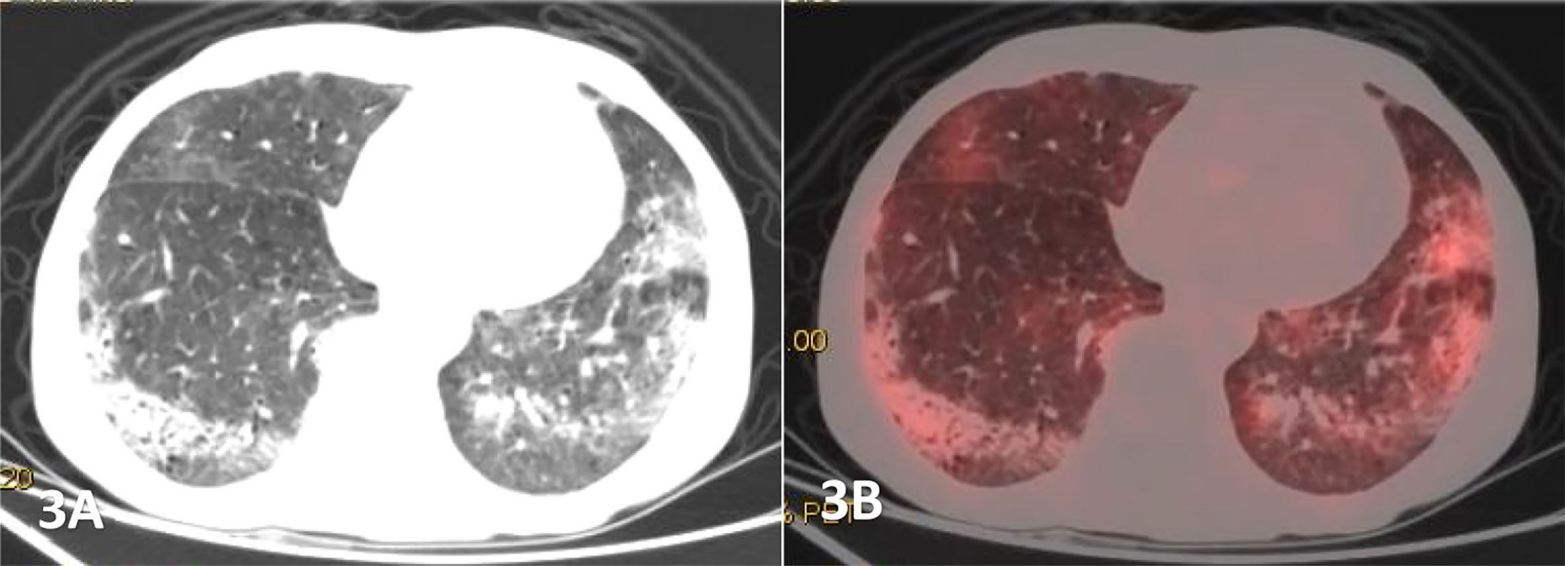
Male, 68 years old, **(A)** was transverse CT, and **(B)** was PET/CT fusion, Diffuse patchy, grid-like increased density shadow was seen in both lungs, which was obvious in two lower lungs with unclear margins. The FDG uptake of the lesions were increased, with a SUVmax of 4.27,and pathological findings of percutaneous lung biopsy were MALT.

**Figure 4:**
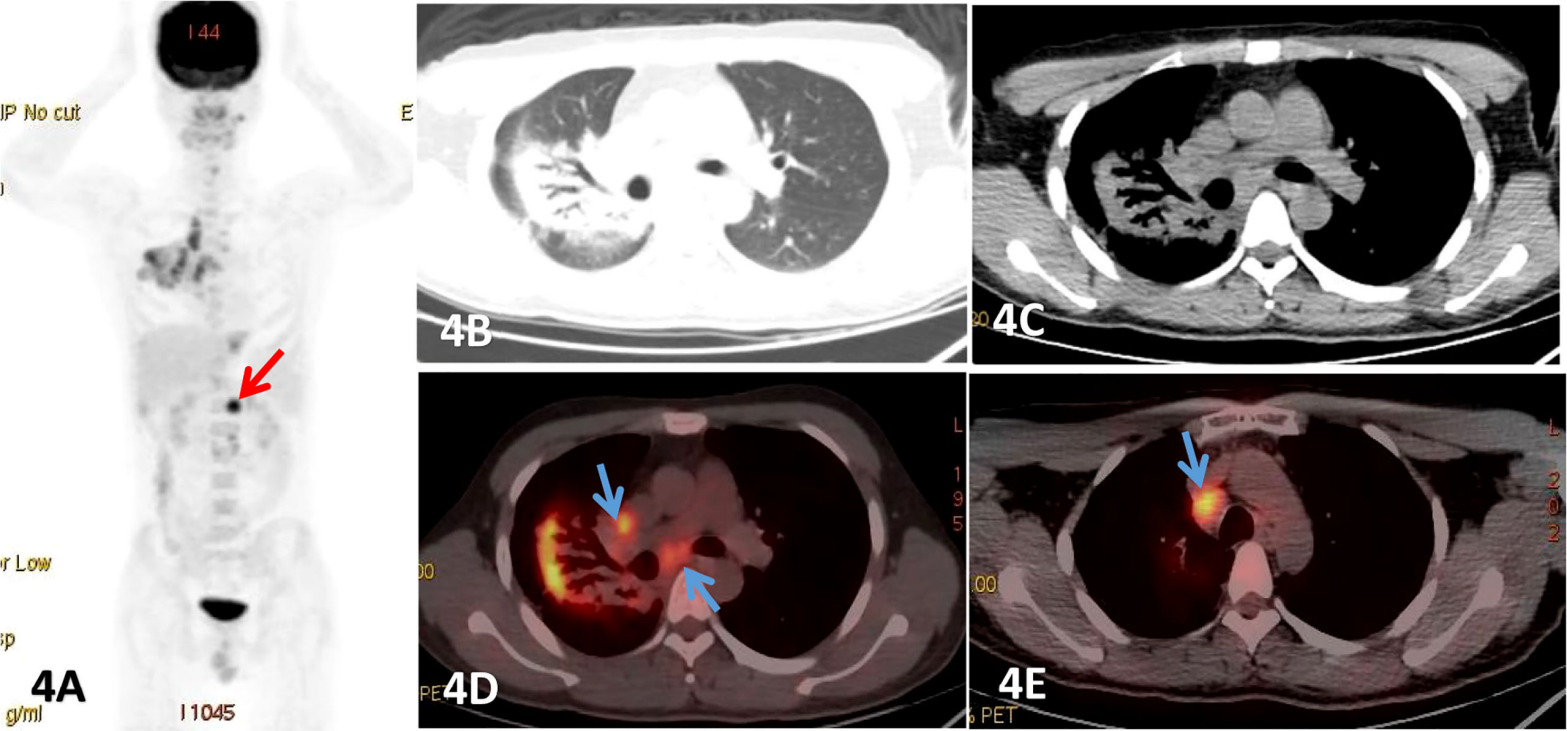
Male, 25 years old, fever, cough, dyspnea for more than 2 months, **(A)** was PET MIP image, **(B, C)** were lung and mediastinum CT transection scan, **(D, E)** were PET/CT fusion image of mediastinum.Patchy consolidation shadows were observed beside the right hilar, and obvious dilated bronchial shadows were observed in the lesions, presenting a translobular distribution with clear margins, which FDG uptake increased with a SUVmax of 8.52, and lung MALT lymphoma was detected by fiberbronchoscopy biopsy. And an enlarged lymph node with significant uptake of FDG was also seen below the diaphragm (red arrow). Endoscopic ultrasonic-guided transbronchial needle aspiration.

Among the 19 patients, 14 (73.7%) were MALT lymphoma which were the most pathological pattern, 3 were DLBCL, and 2 were T-cell lymphoma. The lung was unilateral affected in 14 cases (73.7%), and bilateral affected in 5 cases (26.3%). There were 31 lesions (68.8%) in the right lobe which were much more than those of the left lobe (14 cases, 31.2%). There were 9 cases of single lesion, 7 cases of multiple lesion and 3 cases of diffuse lesion. With regard to CT signs, the air bronchial sign (**Figures 1B**, **2B**, **4B**) was the most common in 11 cases, followed by halo sign and collapse leaf sign in 4 cases each. The lesion was clearly demarcated in 7 cases, and 5 patients had concomitant cystic lesions (**Figure 2A**), including bullae and emphysema. Pleural involvement (**Figure 2B**) occurred in 11 cases, and hilar/mediastinal lymph nodes (**Figures 4D, E**) were present in 6 cases. Based on the predominant radiological features of the 19 patients, the lesions were classified into pneumonic consolidation type (8 cases, **Figures 2**, **4**), node/mass-type (7 cases, **Figure 1**), diffuse interstitial type (3 cases, **Figure 3**) and mixed-type (1 case). The pneumonic consolidation type dominated in MALT lymphoma, and the nodal/mass type and diffuse interstitial type dominated in DLBCL and T-cell lymphoma, respectively. In terms of clinical stage, 8 cases involved only the lung, corresponding to stage IE, and the remaining 11 cases involved other sites, such as hilar/Mediastinal lymphadenopath (5 cases), chest-wall/Diaphragm (2 cases), Subphrenic lymphadenopathy (1 case), as shown in **Table 2**.

**Table 2:** CT manifestations in 20 patients with PPL.

	MALTL (n = 14)	Non-MALTL (n = 5)		Total (n = 19,%)
		DLBCL (n = 3)	T-cell (n = 2)	
Location				
Unilateral	11	3		14 (73.7%)
Bilateral	3		2	5 (26.3%)
Distribution				
Superior lobe of right lung	7	2	2	11 (24.4%)
Middle lobe of right lung	8		2	10 (22.2%)
Inferior lobe of right lung	8		2	10 (22.2%)
Superior lobe of left lung	5		2	7 (15.6%)
Inferior lobe of left lung	4	1	2	7 (15.6%)
Number				
Single lesion	7	2		9 (47.4%)
Multifocal lesions	6	1		7 (36.8%)
Diffuse lesions	1		2	3 (15.8%)
CT features				
Air bronchial sign	9	2		11 (57.9%)
Transleaf sign	3	1		4 (21.1%)
Bronchiectasia	2			2 (10.5%)
Halo sign	2	2	2	6 (31.6%)
With cystic pulmonary lesions	5			5 (26.3%)
Clear-cut margin	7			7 (36.8%)
Pleural invovlement	10	1		11 (57.9%)
Hilar lymphadenopathy		2		2 (10.5%)
Mediastinal lymphadenopathy	1	2	1	4 (21.1%)
Pattern				
Pneumonic consolidation type	7			7 (36.9%)
Node/mass-type	5	3		8 (42.1%)
Diffuse interstitial type	1		2	3 (15.8%)
Mixed-type	1			1 (5.2%)
Stage				
IE lung involvement	8			8 (42.1%)
IIE stage, lung and hilar /Mediastinal lymphadenopathy	3	2		5 (26.3%)
IIEW stage, lung and chest-wall /Diaphragm involvement	2			2 (10.5%)
IIIstage, lung and Subphrenic lymphadenopathy	1			1 (5.2%)
IVstage, Diffuse lesions	1		2	3 (15.8%)

PPL, Primary pulmonary lymphoma.

DLBCL, Diffuse large B-cell lymphoma.

MALTL, Mucosa-associated lymphoid tissue lymphoma.

### 3.3 PET metabolic characteristic

In the 19 patients, the average SUVmax of the lesion was 7.23 ± 4.75, the ratio of SUVmax (lesion/liver) was 3.46 ± 2.25, and ratio of SUVmax(lesion/mediastinal blood pool) was 5.25 ± 3.27. The different pathological types of PPL showed different levels of FDG uptake. The FDG uptake of DLCBL was the highest, whose lesion average SUVmax was 15.33 ± 6.30, ratio of SUVmax(lesion/liver) was 7.96 ± 1.47, and ratio of SUVmax (lesion/mediastinal blood pool) was 11.61 ± 1.49. The FDG uptake of MALT lymphoma and T-cell lymphoma were similar. The lesion average SUVmax, ratio of SUVmax(lesion/liver), ratio of SUVmax (lesion/mediastinal blood pool) of MALT lymphoma were 5.74 ± 2.65,2.61 ± 1.06,3.99 ± 1.81, respectively. And lesion average SUVmax, ratio of SUVmax(lesion/liver),ratio of SUVmax (lesion/mediastinal blood pool) of T-cell lymphoma were 5.50 ± 1.13, 2.63 ± 0.30, 4.60 ± 0.88, respectively.

We compared the lesion’s average SUVmax of the following situations: MALT lymphoma and DLCBL, single lesion and multifoci lesions, pneumonic consolidation type and node/mass-type, with/without air bronchial sign,with/witout pleural involvement,I stage and other stages. It was found that there was significant statistical significance for the lesions SUVmax betweem MALT lymphoma and DLCBL with P-value<0.001. Besides, the lesions SUVmax between pneumonic consolidation type and node/mass-type,I stage and other stages were also statistical significant (P-value<0.05), as shown in **Table 3**.

**Table 3:** PET metabolic characteristic and its relationship of the 19 PPL patients.

	MALTL	DLCBL	T-value	P-value
Lesion average SUVmax	5.74 ± 2.65	15.33 ± 6.30	4.468	<0.001
Ratio of SUVmax(lesion/liver)	2.61 ± 1.06	7.96 ± 1.47		
Ratio of SUVmax(lesion/ mediastinal blood pool)	3.99 ± 1.81	11.61 ± 1.49		
	MALT	T-cell		
Lesion average SUVmax	5.74 ± 2.65	5.50 ± 1.13	0.151	>0.05
Ratio of SUVmax(lesion/liver)	2.61 ± 1.06	2.63 ± 0.30		
Ratio of SUVmax(lesion/ mediastinal blood pool)	3.99 ± 1.81	4.60 ± 0.88		
	Single lesion	Multifocal lesions		
Lesion average SUVmax	8.44 ± 6.12	6.59 ± 3.52	0.71	>0.05
	Pneumonic consolidation type	node/mass-type		
Lesion average SUVmax	4.92 ± 2.66	9.98 ± 5.64	2.16	<0.05
	With air bronchial sign	Without air bronchial sign		
Lesion average SUVmax	7.32 ± 5.97	7.10 ± 2.65	0.098	>0.05
	With Pleural involvement	Without Pleural involvement		
Lesion average SUVmax	7.13 ± 5.69	7.37 ± 3.42	0.116	>0.05
	I stage	II˜IV stages		
Lesion average SUVmax	4.13 ± 1.88	9.48 ± 4.99	2.70	<0.05

## 4 Discussion

### 4.1 Clinical manifestation

Primary pulmonary lymphoma (PPL) is characterized by lymphocyte infiltration in or along the periphery of lymphatic vessels in bronchial mucosa-associated lymphoid tissue (11). It tends to occur in the middle age and older, with a peak age of onset ranging from 50 to 80 years and an average age of 60 years, affecting more males than females (11, 12). PPL is rare in patients younger than under 30, which is often associated with possible immunodeficiency (13, 14). The average age of the 19 patients with PPL enrolled in this study was 59.1 years, and 57.9% of them were over 60 years old, with only one patient younger than 30-years old. We also identified and included more males than females, which is broadly consistent with literature reports. Incidentally, it is still controversial whether there is any gender preference for PPL (15). PPL was overwhelmingly characterized by non-Hodgkin’s lymphoma (NHL), and with its most common subtype mucosa-associated lymphoid tissue lymphoma (MALTL), accounted for approximately 70-90% (16, 17). Likewise, diffuse large B-cell lymphoma (DLCBL) accounted for 10˜20% (7). While lymphomatoid granulomatosis (LG), peripheral T-cell lymphoma, primary pulmonary HL occurs rarely, as we also partly reported in this study. MALTL was predominant (74.5%), followed by DLCBL (15.9%) and peripheral T-cell lymphoma (10.6%), and no other types were found.

The clinical symptoms of enrolled PPL patients in our study were usually mild, and even many MALTL patients have no symptoms (37-50%). Those with symptoms showed only non-specific respiratory symptoms, such as cough, dyspnea, chest pain, fatigue, occasional hemoptysis, etc. Systemic B symptoms (fever, night sweats and weight loss) rarely appeared (18, 19). Most lesions were localized, mostly in stage I or II. However, DLBCL is a high-grade lymphoma with respiratory symptoms, and about 1/3 of patients have systemic symptom B, and the disease often progresses rapidly (20). In this paper, the majority of PPL patients had mild clinical symptoms, mainly cough and fever, with 8 cases each. In fact, 28.6% MALTL patients showed no clinical symptoms, but DLBCL symptoms were relatively severe with fever and respiratory symptoms, and 2 cases had symptom B. The main clinical stages were stage I and stage II, with 8 cases each, accounting for 42.1% and 84.2% in total. In terms of etiology, MALTL is thought to occur secondary to inflammation and auto-immune processes. To mention, MALTL in the lung may be caused by bronchial reactions to various irritating antigens, including smoking and immune diseases (21). About 1/4 of patients have a history of auto-immune diseases, such as rheumatism, Sjogren’s syndrome, and mixed connective tissue diseases, especially Sjogren’s syndrome (22). In association with 16% of primary MALT lymphomas, the incidence of lymphoma was 6.6-44 times higher than that of the general population (22, 23).

PPL could be single or multiple, involving one or both lungs, though it can invade the unilateral lobe (24). Also, the probability of PPL to be affected on the right side is more common compared to the left side (48.9% vs. 43.3%) (25). The cases reported in this paper were mainly unilateral (n=14/19), with mainly single number of lesions. The number of lesions in the right lung (31 cases) were significantly higher than that in the left lung (14 cases), which is consistent with the literature.

### 4.2 Pathology and CT findings

Multislice spiral CT of the chest is the preferred method for evaluating PPL, and thin-slice CT is more valuable for displaying detailed and characteristic images of lesions (26). Tumor cells of PPL can infiltrate and diffuse along the bronchial and vascular peripheral lymphatic sinus pathways, resulting in thickening of tissue structures around bronchial, vascular and lymphatic vessels or local formation of nodules or masses (27). Tumor cells can also infiltrate interlobular fissures and alveolar septa, resulting in interstitial pneumonia-like changes and ground glass changes in lung (28). Infiltration into the alveolar cavity may also lead to acinar nodular changes, resulting in nodules, masses of different sizes and lung consolidation and other manifestations (9).

The CT findings of PPL were found to be diverse, and were divided into 4 types: (A) Nodular/mass type (**Figure 1**): Nodular or mass shadows distributed along the bronchovascular bundle in the lungs, most of which were without pleural effusion, uneven density, and without calcification. (B)Pneumonia consolidation type (**Figure 2**), showed patchy consolidation shadows distributed along lung segments or lobes, with high density in the center and gradually low density in the periphery. (C)Diffuse interstitial type (**Figure 3**): the lesions invaded the lung interstitial and gradually invaded the lung parenchyma, presenting as diffuse patchy distribution along the pulmonary vessels and bronchi, increased density shadow of ground glass, and blurred boundar. (D) Mixed type: mixed with 2 or more pathological types, mostly pneumonia consolidation type and nodular mass type. In low-grade NHL, mass or nodular pneumonia and solitary lobular pneumonia are more common, whereas in high-grade NHL, multilobular lesions are more common (26). In our study, we observed that pneumonia consolidation type (42.1%) and nodular/mass type (36.8%) were the most common morphological pattern. The overall percentages of these two types were 78.9%, followed by diffuse interstitial typemass (15.8%) and mixed type (5.3%).

The common CT signs of PPL mainly include: air bronchial sign, halo sign, transblade sign and bronchiectasia. Air bronchogram is considered as a notable manifestation of PPL, especially in MALT lymphoma, and manifests in 50% of individuals with MALTL (2). A previous retrospective study reported that the main imaging features of PPL were consolidation and ground-glass opacity, accompanied by bronchiectasis (13). In our study, the most common sign was bronchial air sign (n=11/19, 57.9%), followed by halo sign (n=6/19) and leaf collapse sign (n=4/19). The translobular distribution of the lesion, the surrounding ground glass “halo”,and the slightly dilated air bronchial signs were helpful in the diagnosis of PPL. In addition, 11 patients with pleural infiltration were found to be pleural thickening or adhesion. Angiographic signs have also been reported as common concomitant signs of lymphoma (29), however, we excluded this parameter. Other rare imaging manifestations of lung MALT lymphoma included enlargement of mediastinal and hilar lymph node in 30% of patients, and pleural effusion in 10% of patients (30, 31). In our study, 3 cases of hilar lymph node enlargement and 2 cases of mediastinal lymph node enlargement were reported, and 3 patients were found to have pleural effusion. PPL may also be accompanied by multiple cystic changes in the lung. There were 5 cases with cystic foci, of which vascular perforation was the characteristic manifestation with unclear pathological mechanism, which may be secondary to lymphocytic interstitial pneumonia (LIP). It has been reported that 5% of LIP may progress to MALT lymphoma (32).

### 4.3 18F-FDG PET/CT metabolic characteristic

Fluoro-18-fluorodeoxyglucose positron emission tomography/Computer Tomography (18F-FDG PET/CT) is a vital imaging tool, which has widely been used in staging, resetting, and follow-up of various tumors including HL and NHL (33). 18F-FDG PET/CT on the other hand is more representative of dual-mode imaging of anatomy and functional metabolism. It can visualize not only the anatomical structure of lesions, but also evaluate the biological characteristics of tumors from glucose metabolism level, which has certain advantages in the diagnosis, staging and efficacy evaluation of PPL (34, 35).

Pulmonary MALT lymphoma referred to low-grade, extranodal marginal B-cell NHL with low or uneven FDG uptake, and in most cases, there was only a detectable but insignificant increase in FDG uptake. A few studies investigated the metabolic behavior of pulmonary MALTL and found that the detection rate of 18F-FDG PET/CT was very high, with an average SUVmax ranges from 3.3 to 7.5 (36–38). It was discussed that patients with indolent PPL such as MALTL often had slightly increased 18F-FDG uptake with the SUVmax of 0˜6, and the uptake value was unrelated to tumor size (39). However, differences in 18F-FDG uptake s may also arise due to different histological features, e.g., MALT lymphoma with plasmacytic differentiation more frequently show18F-FDG avid lesions. Albano Domenico et al. analyzed 18F-FDG PET/CT imaging data of 28 pulmonary MALTL patients and found that the only variable significantly correlated with 18F-FDG PET/CT positivity was the maximum diameter of the lesion; MALT lung lymphoma with small lesions were less 18F-FDG avid compared with larger lesions (35). In addition, the authors also confirmed the crucial role of tumor size for each type of morphological disease pattern. On the other hand, no correlations were demonstrated between semi-quantitative 18F-FDG PET/CT characteristics and tumor size. Thus, the morphological pattern and other radiological features didn’t seem to influence 18F-FDG avidity. As for DLBCL, it is a high-grade lymphoma with strong aggressiveness, and its FDG uptake is significantly higher than other types. Both DLBCL and MALT can sometimes be seen in histology, making some authors believe that DLBCL can be transformed from low-grade B-cell lymphomas to MALTL, but this view is still controversial.

In our study, all the 19 patients showed increased metabolism, but the uptake degree was not consistent. The SUVmax of lesion ranged from 2.58 to 22.6. The FDG uptake of DLBCL was significantly higher than that of MALT lymphoma (P<0.001). But the FDG uptakes in MALT lymphomas and T-cell lymphomas were quite similar. We also found that the FDG uptake of nodular/mass lesions was higher compared to pneumonia consolidation, while the FDG uptake of clinical I stage PPL was found to be lower than that of other stages, with statistically significant differences (P<0.05). One study reported no significant differences between positive and negative 18F-FDG PET/CT when comparing the metabolic and morphological pattern of presentation, presence of bronchiectasis and air bronchogram (35), which is consistent with our results. In addition, a very important feature of 18F-FDG PET/CT is that whole-body imaging is possible with a single scan and comprehensive information can be obtained, which is conducive to the detection of extrapulmonary lesions and plays an important role in the staging of PPL. Moreover, the different stages have different treatment methods, for example, IE˜II1E PPL is mainly treated with surgery (25). In the current study, one patient was found to have subphrenic lymph nodes with high metabolism, which raised the stage from IE to III (**Figure 4**). In the other 2 mediastinal lymph nodes, although their diameter was less than 10-mm, but 18F-FDG PET/CT metabolism was significantly increased with a SUVmax of 6.2. In addition, the postoperative pathology confirmed the infiltration that also changed the stage from IE to IIE. Furthermore, 18F-FDG PET/CT has been shown to be considerably more accurate than anatomical imaging in assessing response, as it is able to distinguish between viable tumor and nonviable post treatment findings and can identify individuals with treatment resistance to certain forms of chemotherapy at an early stage, providing the clinicians with a sufficient time window to modify the therapeutic strategy (40, 41).

It is necessary to distinguish PPL from pneumonia, lung cancer, or tuberculosis. As lung cancer patients exhibited severe respiratory symptoms, irregularly thickened bronchial wall, narrow or interrupted bronchial lumen, increased in lung cancer-related tumor markers such as CEA and SCC, and usually a higher FDG uptake. In case of peripheral lung cancer, the fine burr, pleural depression, vascular aggregation and other signs in the edge were well known (8, 42). In lobar pneumonia, lamellar consolidation shadows were evident, the bronchial wall was usually not thickened, the bronchial lumen occured as relatively unobtruded, and hilar and mediastinal lymph nodes generally were not enlarged. It could be absorbed after anti-inflammatory treatment. The total number of white blood cells and neutrophils were elevated, and FDG uptake was also increased, but more uniformly (43). The pulmonary tuberculosis was found to be located mainly in the apicoposterior segment of the upper lobe and the dorsal segment of the lower lobe with satellite foci around the lesion. Both calcification and necrosis were also seen in some lesions, and the tuberculin test was positive (8, 42, 44). Nevertheless, PPL patients often had periodic fever, mild symptoms, air bronchial signs were common in the lesions, bronchial lumen was unobstructed, and the bronchial wall was uniformly thickened in the lesion. Moreover, anti-inflammatory therapy was ineffective, and blood routine was mostly normal with no increase of tumor markers and satellite lesions. The 18F-FDG uptake was often moderately elevated. Certainly, 18F-FDG uptake and morphological manifestations of these diseases may overlap, which should be comprehensively analyzed in combination with clinical features and response to treatment (39).

Studies also reported about that 18F-FDG PET/CT images of PPL had certain characteristics, they were not specific and the clinical symptoms were not typical, so the diagnosis was still difficult (6, 7, 45). In some reports, PPL has also been considered as pneumonia or lung cancer, tuberculosis and even diffuse interstitial lung disease (29). In our study, only 3 cases of PPL were correctly diagnosed on 18F-FDG PET/CT, and the remaining 16 cases were inflammatory lesions (lobar pneumonia 6 cases, tuberculosis and diffuse interstitial pneumonia 3 cases in each), or lung cancer (4 cases). Thus, the misdiagnosis rate was as high as 84.2%. To date, the pathological examination remains the gold standard for PPL diagnosis. Therefore, in similar inflammatory lesions with unknown diagnosis or ineffective treatment, bronchoscopy or CT-guided lung biopsy should be considered at the earliest opportunity to clarify the nature of the lesions. A cautious approach is also warranted as bronchoscopy is often negative and percutaneous lung biopsy has a high diagnostic rate. In this regard, most cases in our study were diagnosed by percutaneous lung biopsy (10 cases), while only 4 cases were diagnosed by bronchial biopsy. Clearly, the success rate of biopsy can be significantly improved under the guidance of 18F-FDG PET/CT, because 18F-FDG PET/CT can identify the site of tumor activity due to the heterogeneity of the tumor. Following this, we also obtained reliable specimen from the 10 percutaneous lung biopsy cases under the guidance of 18F-FDG PET/CT.

Of course, there are also certain limitations of our study, such as, being single center research, the bias of patient selection and the limited number of patients are inevitable issues. We also did not conduct further investigations to determine the role of 18F-FDG PET/CT in evaluating the curative efficacy of PPL. Importantly, the number of patients in each subgroup of the PPL was very limited. In particular, DLCBL and T-cell lymphoma were observed in only 2-3 cases. However, we intend to analyze and summarize additional subgroups once we have collected more cases.

## 5 Conclusion

Although the imaging manifestations of PPL were diverse, non-specific, and the clinical symptoms were not typical, making the diagnosis difficult and subject to easy misdiagnosis. Still, the morphology and metabolism of PPL on 18F-FDG PET/CT images showed certain features that may provide a better understanding of PPL disease. In addition, 18F-FDG PET/CT whole-body imaging showed the potential to refine the staging of PPL. Importantly, functional 18F-FDG PET/CT imaging readily reflected tumor cell activity, thus allowing the selection of an optimal biopsy site.

## Data availability statement

The original contributions presented in the study are included in the article/supplementary material. Further inquiries can be directed to the corresponding author.

## Ethics statement

The studies involving human participants were reviewed and approved by Ethics Committee of Jiangxi Provincial People’s Hospital. The patients/participants provided their written informed consent to participate in this study.

## Author contributions

Conceptualization: YP, WQ, FL. Data collection and interpretations: YP. Data curation: ZL, QZ, YH, YW, AS, IS-W. All authors contributed to the article and approved the submitted version.

## Conflict of interest

The authors declare that the research was conducted in the absence of any commercial or financial relationships that could be construed as a potential conflict of interest.

## Publisher’s note

All claims expressed in this article are solely those of the authors and do not necessarily represent those of their affiliated organizations, or those of the publisher, the editors and the reviewers. Any product that may be evaluated in this article, or claim that may be made by its manufacturer, is not guaranteed or endorsed by the publisher.

## Abbreviations

18F-FDG PET/CT, Fluoro-18-fluorodeoxyglucose positron emission tomography/Computer Tomography; CT, Computer Tomography; PPL, Pulmonary primary lymphoma; DLBCL, Diffuse large B-cell lymphoma; MALTL, Mucosa-associated lymphoid tissue lymphoma.
